# Excision and reconstruction of dorsal nasal mucous cyst using dorsal nasal flap technique: a case report and literature review

**DOI:** 10.1093/jscr/rjad667

**Published:** 2023-12-14

**Authors:** Mohamed B Ahmed, Fatima S Al-Mohannadi, Khaled E Elzawawi, Abeer Alsherawi, Mahmoud Althalathini

**Affiliations:** Plastic Surgery Department, Hamad General Hospital, Hamad Medical Corporation, Doha, Qatar; College of Medicine, QU Health, Qatar University, Doha, Qatar; Plastic Surgery Department, Hamad General Hospital, Hamad Medical Corporation, Doha, Qatar; College of Medicine, QU Health, Qatar University, Doha, Qatar; Plastic Surgery Department, Hamad General Hospital, Hamad Medical Corporation, Doha, Qatar; Plastic Surgery Department, Clinica Joelle, Qatar

**Keywords:** rhinoplasty, mucous cyst, dorsal nasal flap, complications

## Abstract

Congenital nasal masses are very rare presentations. Among these masses is a mucous cyst, which might be considered either a congenital or an acquired mass. Our report presents a case of recurrent dorsal nasal swelling that was initially managed with an open rhinoplasty. However, 1 year after the surgery, the swelling started to grow rapidly, and the patient presented with a disfigured nose. We scheduled the patient for the excision of the dorsal nasal swelling and reconstruction using the dorsal nasal flap approach. Several surgical techniques have been performed and published in the literature; however, this is the first time the dorsal nasal flap technique is being reported as a surgical approach to dorsal nasal mucous cysts. While mucous cyst formation might be congenital, the majority occur after rhinoplasty surgery. However, they can be prevented by minimizing unnecessary trauma during the surgery and ensuring the thorough removal of all epithelial tissue and foreign bodies.

## Introduction

Congenital nasal masses are rare entities that have an incidence of 1/2000–4000 births. These include mucous cyst (MC), dermoid cysts, polyps, gliomas, and encephaloceles [1]. However, the differentials can consist of traumatic, benign, or malignant lesions, vascular lesions, and inflammatory masses [2]. The most common congenital nasal anomaly is dermoid sinus cysts, accounting for 1%–3% of all types of dermoid cysts. In addition, the incidence in children is around 61%. Therefore, it is usually diagnosed in the first 3 years of life. Nonetheless, in some cases, diagnoses can be made later in life, resulting in recurrent airway infections, airway obstruction, and, in severe cases, intracranial complications [1].

MC is a type of nasal anomaly that can be either congenital or acquired. Acquired MC mostly occurs following rhinoplasties. They are expandable cystic lesions lined with epithelium and filled with mucus [3]. In addition, the most common site of MC formation is the dorsum, but it still can appear at other regions of the nose [4]. The first case of MC formation following rhinoplasty was first reported by McGregor *et al*. [5], and many cases have been documented ever since. On the other hand, congenital dorsal nasal MC is very rare with only one case published in the literature [6].

In this paper, we present a case of excision and reconstruction of a recurrent dorsal nasal MC using dorsal nasal flap technique.

## Case presentation

This is a case of a 33-year-old Nepalese gentleman who presented to our clinic in October 2020 with the chief complaint of a recurrent large nasal swelling that had persisted for 1 year. He had a history of small nasal swelling that first appeared 7 years ago with very slow progression in size. The patient denied any previous facial trauma or surgeries. Two years ago, he had undergone surgical removal of the swelling through an open rhinoplasty with a columellar incision in Nepal. However, the swelling started to appear and grow again 1 year after the surgery. By the end of the 2nd year, the swelling continued to enlarge, leading to both patient embarrassment and disfigurement of the nose.

**Figure 1:**
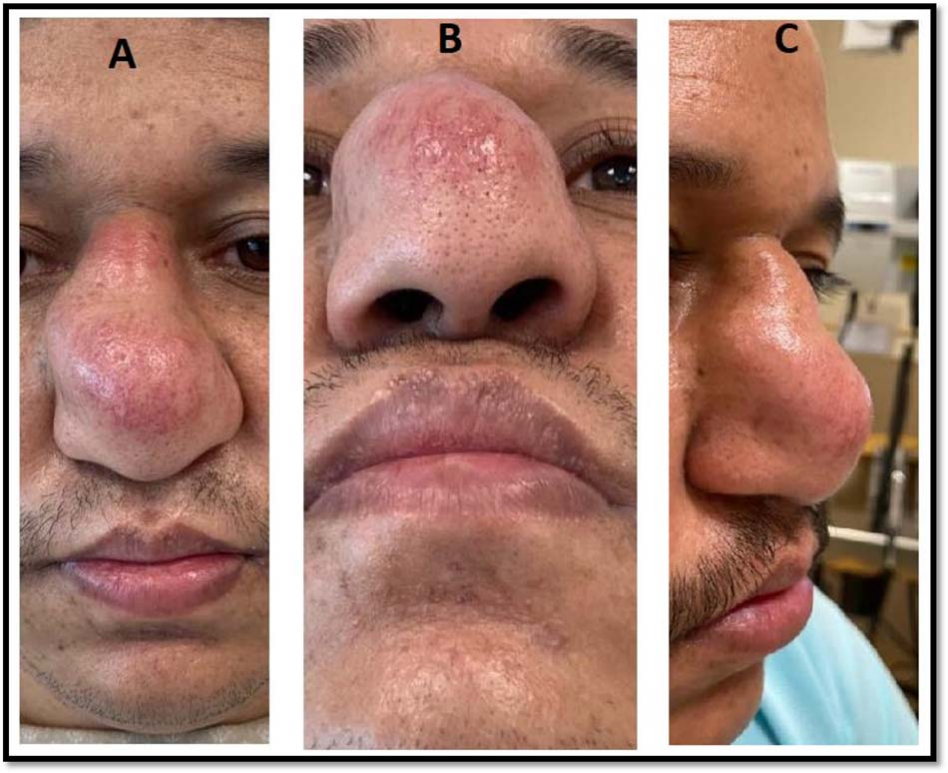
Preoperative picture of the patient’s nasal dorsal swelling.

**Figure 2:**
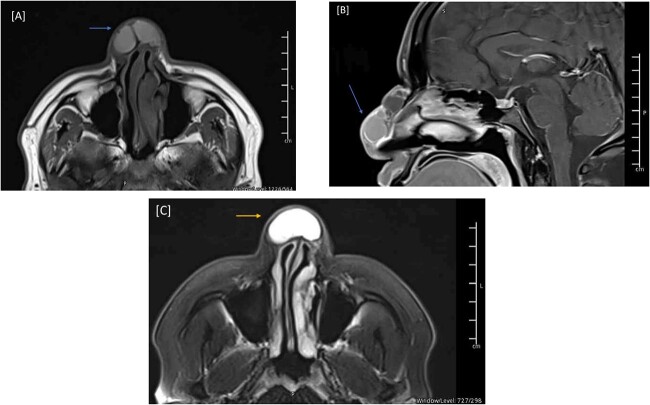
MRI of the head, and paranasal sinuses (Pre-Op). (A) There is a lobulated multilocular cystic lesion seen at the dorsum of the nose, some of the cysts are showing bright signal intensity in T1 (arrow) and T2/FLAIR (C) (arrow) representing high proteinaceous content. No connection to the cranial cavity, features are likely presenting dermoid cyst. (B) The lesion shows peripheral postcontrast enhancement. No diffusion restriction.

During the physical examination, there was a large globular swelling on the dorsum of the nose extending to both sides with erythema but without warmth (Fig. 1). The patient’s breathing was not affected. A magnetic resonance imaging (MRI) of the head with contrast showed a multiloculated midline cystic lesion on the dorsum of the nose, with some parts showing high proteinaceous content. No connection to the cranial cavity was observed (Fig. 2). These features suggest a dermoid cyst. The size of the lesion is depicted in Supplementary Fig. S1.

**Figure 3:**
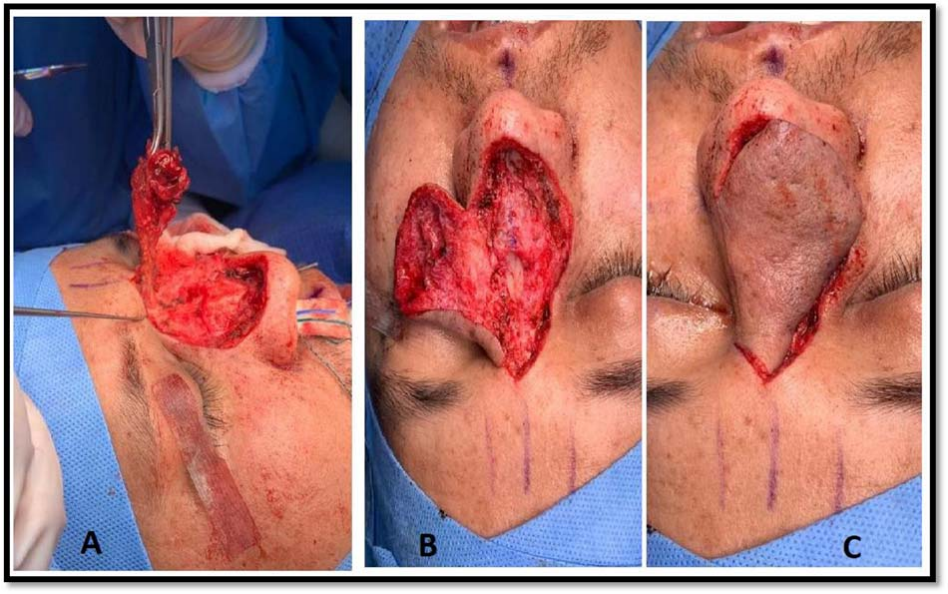
Intraoperative picture of the patient’s nasal dorsal swelling. Panel (A) shows the swelling before excision. Panel (B) shows the dorsum of the nose after swelling excision. Panel (C) shows the raised dorsal nasal flap.

As a result, the patient was scheduled for the excision of the dorsal nasal mass, employing a dorsal nasal flap approach. A complete excision of the swelling was performed from the caudal nasal dorsum up to the radix of the nose (Fig. 3). In addition, tip refinements were performed to achieve a better esthetic outcome as well as functional improvement. We opted for the dorsal nasal flap approach because it provides good exposure, especially since the swelling extended over the dorsum and reached the radix. In addition, the patient’s history of open rhinoplasty led us to anticipate severe adhesions.

The surgical pathology report revealed that the nasal mass biopsy consisted of a cyst lined with respiratory epithelium, and the sole content within the nasal mass was mucin. The detailed description showed a cystic lesion with its cyst wall lined by ciliated respiratory type epithelium. The cyst wall was variably fibrotic with sparse chronic inflammation and granulation tissue. No cytologic atypia was identified. The aforementioned findings are consistent with MC.

**Figure 4:**
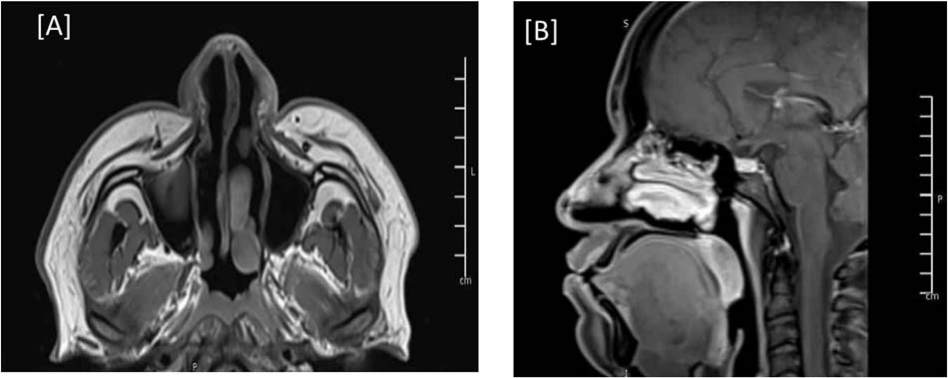
MRI of the head, and paranasal sinuses (post-op). (A, B) Axial and Mid-sagittal T1 sequence showing total resection of the external nasal mass lesion with a heterogenous enhancement seen likely postoperative changes. No obvious residual mass lesions can be seen.

**Figure 5:**
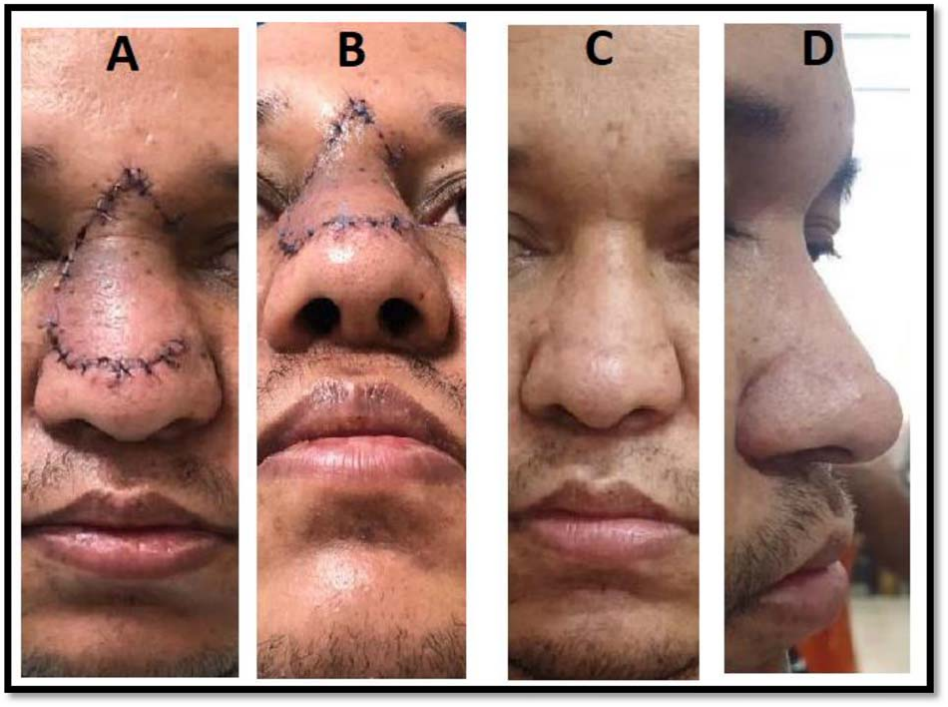
Postoperative pictures showing the short-term outcome of the procedure (A, B) and the long-term outcome (C, D).

The patient was discharged three days after the surgery with a clean, dry wound and no signs of infection or hematoma collection. The flap was viable with good color and normal capillary refill time. After discharge, the patient had regular follow-ups to assess the wound status and the flap viability. The patient expressed happiness with the shape of his nose. Three months post-op, a follow-up MRI showed total resection of the nasal lesion with no evidence of residuum or recurrence (Fig. 4). The patient was followed in our clinic for 14 months with no signs of recurrence (Fig. 5). During his last visit, he expressed his happiness regarding the relief of symptoms and the shape of his nose.

## Discussion

Dorsal nasal MC could be either a congenital or an acquired lesion. Congenital dorsal nasal MC are exceptionally uncommon conditions. The most common differential diagnoses for this lesion are gliomas, dermoid cysts, and encephaloceles. In the literature, only one case has been reported of a 1-month-old male baby with a congenital dorsal nasal MC primarily located on the tip of the nose [6]. A majority of the dorsal nasal MC cases published in the literature are acquired and are mainly considered among the late period complications of rhinoplasty surgery.

The most common location of MC is along the nasal dorsum near nasal osteotomy lines, but it can also appear in other areas [4, 7, 8]. Before confirming MC diagnosis, multiple differential diagnoses have to be excluded, such as neoplastic lesions, encephaloceles, gliomas, lipomas, osteomas, and dermoid cysts. Therefore, comprehensive history taking, physical examination, nasal endoscopy, and radiographic studies will help to narrow down the differential diagnosis [9].

Many hypotheses have been published in an attempt to explain this phenomenon. However, as of now, no consensus has been reached [5, 10–12].

Many surgical approaches have been discussed in the literature, including the open rhinoplasty approach, the intranasal approach, endoscopic excision, and direct external cutaneous removal [12, 13]. Regardless of the approach used, the main aim is to completely excise the MC with its capsule to ensure absence of recurrence.

In our case, the patient presented with a history of nasal swelling that had started to appear 7 years before his presentation to our clinic. The swelling was growing slowly. No history of trauma or previous surgeries in this area was reported by the patient. He had undergone excision of the swelling in his country 2 years before his presentation to us. One year after the surgery, the swelling started to reappear and increased in size at a faster rate. When he presented to our clinic, the swelling was substantial, causing disfigurement, and was located superiorly on the nasal dorsum. Therefore, a dorsal nasal flap incision approach was used to excise and reconstruct nose. This technique provided us with great exposure and enabled the complete excision of the MC along with its capsule.

In addition, the reconstruction of the nose, along with tip refinement, resulted in excellent functional and esthetic outcomes, leaving both the patient and the surgical team happy and satisfied. Considering the patient’s medical history and pathology findings, we assume that this is a delayed presentation of a congenital dorsal nasal MC. In addition, the recurrence of this lesion after excision is primarily attributed to the limiting approach used in the previous surgery, as the MC was not completely excised. Therefore, we recommend using this approach to excise dorsal nasal masses that extend to the radix. To the best of our knowledge, there is no existing publication in the literature describing such a lesion occurring in an adult and managed using the dorsal nasal flap approach technique.

Therefore, congenital nasal MC should be considered among the differential diagnoses when assessing nasal masses. Although the majority of congenital nasal masses present early in life, delayed presentation should also be considered.

In conclusion, dorsal nasal MC formation is relatively rare and can manifest as either a congenital or an acquired lesion. We recommend employing dorsal nasal flap approach to excise both congenital and acquired dorsal nasal MC, particularly when the cyst extends to the radix.

## Supplementary Material

Fig_1_Supplementary_rjad667

rjad667_dr_30_rjad667
