# Supplementary material for: Excision and reconstruction of dorsal nasal mucous cyst using dorsal nasal flap technique: a case report and literature review
Source: J Surg Case Rep. 2023 Dec 14;2023(12):rjad667. doi: 10.1093/jscr/rjad667 (PMC10725821; doi:10.1093/jscr/rjad667)
Supplement: Fig_1_Supplementary_rjad667 [file fig_1_supplementary_rjad667.docx]

Fig 1 Supplementary. Mid-sagittal and axial measurements of the dorsal nasal swelling preop.


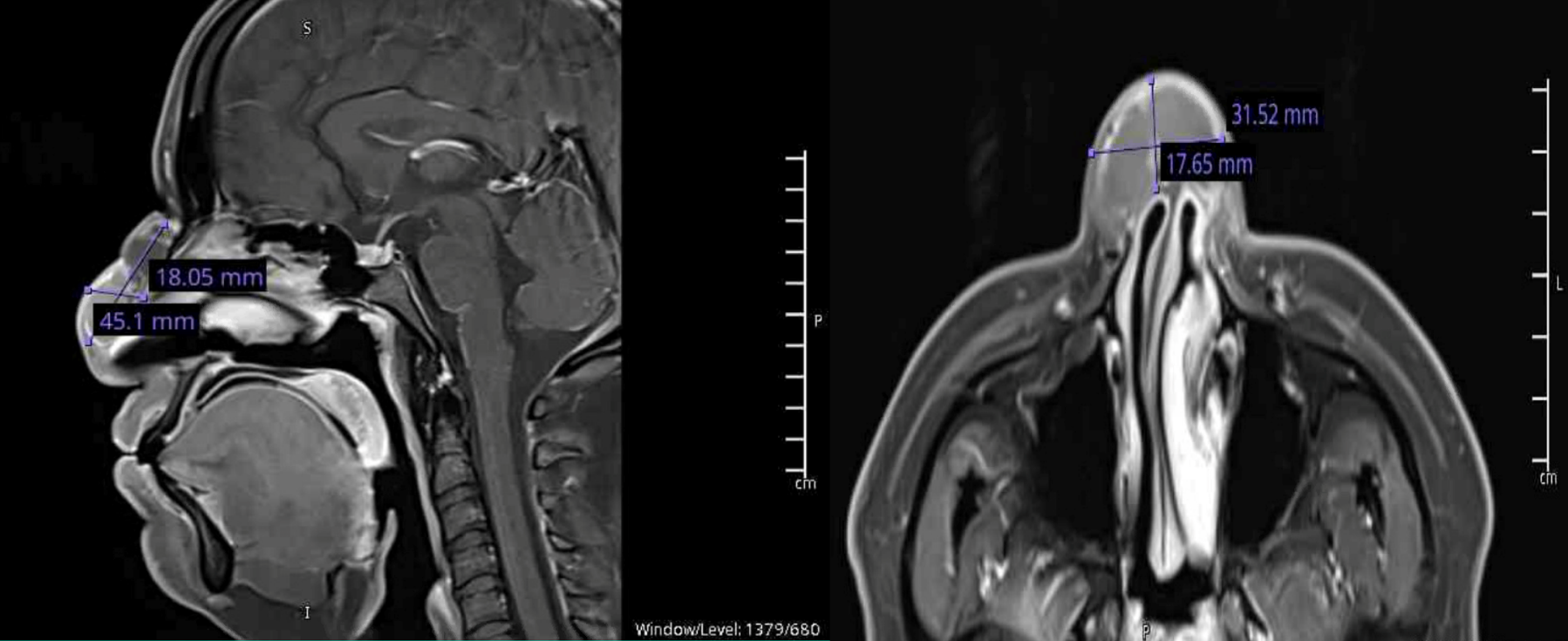


Fig 1 Supplementary.
